# Design of Grating Al_2_O_3_ Passivation Structure Optimized for High-Efficiency Cu(In,Ga)Se_2_ Solar Cells

**DOI:** 10.3390/s21144849

**Published:** 2021-07-16

**Authors:** Chan Hyeon Park, Jun Yong Kim, Shi-Joon Sung, Dae-Hwan Kim, Yun Seon Do

**Affiliations:** 1 School of Electronics Engineering, Kyungpook National University, 80, Daehak-ro, Daegu 41566, Korea; time0117@knu.ac.kr; 2 School of Electronic and Electrical Engineering, Kyungpook National University, 80, Daehak-ro, Daegu 41566, Korea; rhawns4567@knu.ac.kr; 3 Division of Energy Technology, Daegu Gyeongbuk Institute of Science and Technology (DGIST), Daegu 42988, Korea; sjsung@dgist.ac.kr (S.-J.S.); monolith@dgist.ac.kr (D.-H.K.)

**Keywords:** photovoltaics, thin CIGS solar cells, surface passivation, aluminum oxide

## Abstract

In this paper, we propose an optimized structure of thin Cu(In,Ga)Se_2_ (CIGS) solar cells with a grating aluminum oxide (Al_2_O_3_) passivation layer (GAPL) providing nano-sized contact openings in order to improve power conversion efficiency using optoelectrical simulations. Al_2_O_3_ is used as a rear surface passivation material to reduce carrier recombination and improve reflectivity at a rear surface for high efficiency in thin CIGS solar cells. To realize high efficiency for thin CIGS solar cells, the optimized structure was designed by manipulating two structural factors: the contact opening width (COW) and the pitch of the GAPL. Compared with an unpassivated thin CIGS solar cell, the efficiency was improved up to 20.38% when the pitch of the GAPL was 7.5–12.5 μm. Furthermore, the efficiency was improved as the COW of the GAPL was decreased. The maximum efficiency value occurred when the COW was 100 nm because of the effective carrier recombination inhibition and high reflectivity of the Al_2_O_3_ insulator passivation with local contacts. These results indicate that the designed structure has optimized structural points for high-efficiency thin CIGS solar cells. Therefore, the photovoltaic (PV) generator and sensor designers can achieve the higher performance of photosensitive thin CIGS solar cells by considering these results.

## 1. Introduction

The focus on solar cell technology as an ecofriendly future energy generation has increased because of climate change from fossil-fuel generation and potential threats from nuclear power plants [1]. A solar cell is one of the most effective ways to produce electricity among renewable energy. Furthermore, it is of interest in a variety of sensor applications, such as self-powered sensors [2], photodetectors [3], and switchable photovoltaic sensors for machine vision [4]. The solar cell is a multilayered structure consisting of a light-absorbing layer between two electrodes. This absorber layer is critical in creating a photovoltaic (PV) effect that absorbs light and converts it into electrical energy. Various absorbers, such as silicon [5], compound semiconductors [6,7], and organic materials [8] have been developed to produce solar cells that reach higher power-conversion efficiencies and lower the capital investment costs. Solar cells with compound semiconductors for the active layer can achieve high radiation resistance [9] and efficiency because of the advantage of direct bandgap materials [10,11]. Particularly, Cu(In,Ga)Se_2_ (CIGS), a compound semiconductor, is considered a promising material for thin-film solar cells because of its high absorption coefficient in the visible spectrum of sunlight [12,13], high stability [14], flexibility [15], and adjustable bandgap from 1.01 eV to 1.67 eV [16,17,18]. In addition, this material was utilized as a photosensitive solar cell layer of sun sensor for space applications due to its remarkable radiation hardness [19,20,21].

Recently, studies on reducing the thickness of the CIGS absorber layer were conducted to lower the cost [22]. The most critical problem is that the solar cell’s efficiency simultaneously drops as its thickness decreases [22,23]. The insufficient thickness of the active layer lowers the absorption of incident sunlight. Another efficiency loss comes from strong carrier recombination occurring at the rear interface between the molybdenum (Mo) emitter and thin CIGS absorber layer [24,25]. A substantial number of carriers are recombined at the rear interface, thinning the CIGS active layer because of the short lifetimes and diffusion lengths of carriers. To suppress this carrier recombination, a passivation layer with local contacts is inserted into the rear interface, effectively reducing the direct contact area between Mo and CIGS [24,25,26]. Furthermore, it can increase the internal light reflection of the rear contacts, absorbing a larger amount of light into a thin CIGS layer [25,27]. However, large distances between the local contacts increase the mean path of the hole and contact resistance, countervailing the gain from the passivation [28]. To obtain the best efficiency of solar cells with the passivation layer, the passivation coverage and the size of local contacts are the most critical to consider. Thus, the correlation between performance and structure of the passivation layer with nano-micro-scaled structural factors must be investigated.

In this study, we investigate the optical and electrical characteristics of thin CIGS solar cells with a one-dimensional grated thin passivation layer. The dielectric materials could be used as a passivation layer for CIGS solar cells. These materials can improve rear reflectivity compared to an unpassivated surface [29]. This increases the amount of light absorbed at the CIGS layer. Among these various materials, aluminum oxide (Al_2_O_3_) has been most studied as a passivation with local contact in CIGS solar cells [30]. Using atomic layer deposition, the thin Al_2_O_3_ film can be uniformly formed on the Mo layer by precisely controlling the thickness. This thin film remains intact after the CIGS layer is formed at 500 °C. Compared to the unpassivated CIGS rear surface, the Al_2_O_3_ passivation layer reduces about 35% of interface defect density. In addition, the negative fixed charges in Al_2_O_3_ suppress recombination at the CIGS surface through a field effect that reduces the minority charge carrier concentration at the back contact [31]. The optoelectrical simulations were performed by controlling the local contact opening width (COW) and pitch of a grating Al_2_O_3_ passivation layer (GAPL). The optimized structural factors of the GAPL structure showed a higher fill factor (FF) and power-conversion efficiency compared to the unpassivated thin CIGS solar cell. Herein, this study suggests a way to realize high-efficiency thin solar cells for PV generators and sensors.

## 2. Designs of Optimized Thin CIGS Solar Cells with the GAPL

**Figure 1:**
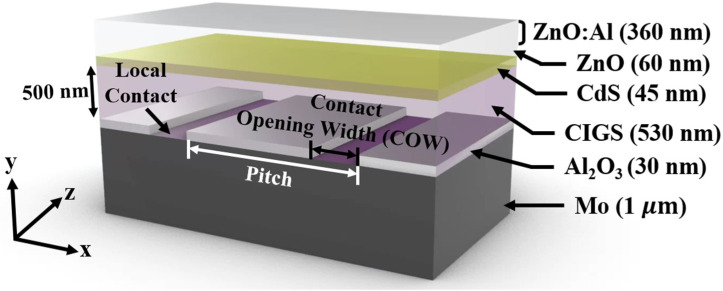
Schematic diagram of a thin CIGS solar cell with the GAPL and its thickness.

The thin CIGS solar cells with the GAPL were designed to investigate the correlation between the structural factors of grating passivation and power-conversion efficiency. Figure 1 shows the structure of the solar cell designed in simulation and the thickness of each material. Each material constituting the thin CIGS solar cells was composed of Mo, Al_2_O_3_, and CIGS with a 0.42 Ga/(Ga + In) ratio, and cadmium sulfide (CdS), zinc oxide (ZnO), and aluminum-doped ZnO (ZnO:Al), with 1 μm, 30 nm, 530 nm, 45 nm, 60 nm, and 360 nm thicknesses, respectively. The Al_2_O_3_ passivation film, which is a diffraction grating structure, was used to construct the local contact surfaces between the CIGS and Mo films. This local contact creates a contact surface with the electrode to allow current to flow, whereas the Al_2_O_3_ reduces current loss by depressing carrier recombination at the CIGS–Mo interface. The ZnO thin film prevents shunt paths in the solar cell because of its high resistivity [32]. Furthermore, to secure transparency to the solar spectrum and conductivity to the minimized resistive loss, the ZnO:Al thin film was formed over the ZnO thin film.

Using the 2D finite-difference time-domain (FDTD) method (FDTD Solutions, Lumerical Inc., Vancouver, BC, Canada), we first obtained power absorbed by each layer from the vertically incident air mass 1.5 (AM1.5) sun spectrum source irradiated with a plane wave of 300 nm to 1200 nm. To represent the infinite plane, the boundary conditions were set to be periodic for the *x*-axis and a perfectly matched layer for the *y*-axis. Second, to estimate the J-V characteristics and efficiency, the calculated power absorption data were imported to the 2D finite element Poisson/draft-diffusion method (CHARGE Solver, Lumerical Inc., Canada). ZnO:Al was used as a top electrode and fixed at 0 V, and the current density was measured by increasing 0.01 V sequentially from 0 V to the opposite Mo electrode as an electrical boundary condition in the CHARGE Solver. Furthermore, the surface recombination velocities for the Mo–CIGS interface, CIGS–CdS interface, and the rest of the interfaces were set to 10^7^ cm/s, 10^4^ cm/s, and 0, respectively [33]. The detailed refractive indices and material properties of Mo [33,34], Al_2_O_3_ [35,36], CIGS [33,37], CdS [33], ZnO [33,38,39], and ZnO:Al [33,40] are referred to in other literature. The CIGS solar cells with a GGI of 0.42 showed higher efficiency than those with a GGI of 0.18, 0.29, 0.5, and 0.64, respectively [33]. Hence, the bandgap of CIGS was set to 1.24 eV with a GGI of 0.42 in the simulation.

## 3. Results and Discussion

**Figure 2:**
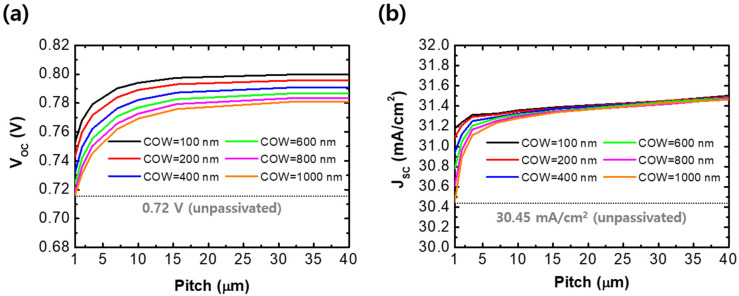
Calculated results of (**a**) open-circuit voltage (V_OC_) and (**b**) short-circuit current (J_SC_) as a function of a pattern pitch with varying GAPL local contact opening widths (COWs). Gray dotted lines express V_OC_ and J_SC_ for an unpassivated solar cell.

To improve the thin CIGS solar cell’s performance and demonstrate its tendency, we analyzed the optical and electrical characteristics of the solar cell by varying the GAPL structure using the FDTD and CHARGE simulations. Figure 2 shows the electrical properties of solar cells calculated using the GAPL pitch and COW. Figure 2a shows the change in the calculated open-circuit voltage (V_OC_) according to the GAPL pattern pitches and local COWs. The passivated structures had a significantly higher V_OC_ than the unpassivated solar cell, which had that of 0.72 V (dotted line). This is due to the reduced carrier recombination velocity at the rear surface of the GAPL [24,31]. As the pitch increased in each COW, the V_OC_ values were saturated after the increments. When the pitch increased from 1 μm to 10 μm with 100 nm of COW, V_OC_ increased from 0.753 V to 0.794 V. The V_OC_ increased to 0.8 V when the pitch was 40 μm, indicating that the carrier recombination velocity decreases because the Al_2_O_3_ proportion at the rear surface increases as the pitch increases, and this velocity converges from a specific pitch. Furthermore, when the COW was 100 nm, the V_OC_ value was the largest in the overall pitch. The maximum value appears because the direct contact area and recombination rate between Mo and CIGS decrease as the COW decreases.

Figure 2b shows the change in short-circuit current (J_SC_) calculated using the GAPL pitch and COW. Compared to the unpassivated solar cell with a J_SC_ of 30.45 mA/cm^2^ (dotted line), the J_SC_ of all passivated solar cells was higher. This is because the reflectivity at the rear surface is improved from the GAPL structure, and the amount of light absorbed in the thin CIGS layer is increased. Similar to the V_OC_ graph, the J_SC_ improved and then saturated as the pitch extended. As the pitch increased, the amount of change in J_SC_ affected from the COW decreased. When the COW increased from 100 nm to 1 μm with a pitch of 1 μm, J_SC_ decreased from 31.2 mA/cm^2^ to 30.45 mA/cm^2^. However, the difference shrank to 0.04 mA/cm^2^ when the pitch extended to 40 μm. Although the increased pitch with the reduced COW improves the reflectivity and depresses the carrier recombination at the rear surface, the amount of reflected light and reduced recombination carriers reached their limits. Furthermore, when the pitch is smaller than 10 μm, J_SC_ is more sensitive to the size of the nano-scaled COW.

**Figure 3:**
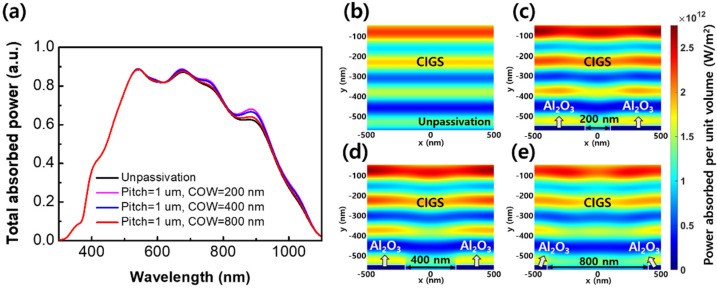
Simulation results of absorbed powers for an unpassivated thin CIGS solar cell and the thin solar cells with the grating Al_2_O_3_ passivation layer (GAPL). (**a**) The spectral characteristics of the total absorbed power in the solar cells without the passivation layer and with COWs of 200 nm, 400 nm, and 800 nm in the same pitch of 1 μm. (**b**–**e**) The spatial profiles for the power absorbed per unit volume with respect to the *xy*-plane at a wavelength of 886 nm. (**b**) The unpassivated solar cell. (**c**–**e**) The solar cells with COWs of 200 nm, 400 nm, and 800 nm, respectively.

Figure 3 shows the variation in optical characteristics of an unpassivated thin CIGS solar cell and thin CIGS solar cells with COWs of 200 nm, 400 nm, and 800 nm in the same pitch of 1 μm. Figure 3a shows the spectral characteristics of the total absorbed power in an unpassivated solar cell and solar cells with COWs of 200 nm, 400 nm, and 800 nm. By the high reflectivity at the rear surface between Al_2_O_3_ and CIGS, the amount of absorbed power in the active layer was higher in solar cells with the GAPL than in the unpassivated solar cell. In the infrared wavelength region, the light absorption increased as the GAPL COW decreased. In particular, when the COW was reduced from 800 nm to 200 nm, the total absorbed power increased from 0.64 to 0.68 in a wavelength of 886 nm. To verify the light absorption for a CIGS layer with the GAPL, the average spectral absorbed power was calculated in the overall spectrum. The average spectral absorbed power in the solar cell with a COW of 800 nm was calculated as approximately 0.490. Furthermore, the solar cell with a 200 nm COW shows a 1.84% improvement on the average absorbed power, which was calculated as 0.499.

To investigate the optical effect from the variations in the GAPL COW, the spatial profiles for the power absorbed per unit volume with respect to the *xy*-plane of the solar cells were analyzed using numerical simulation using the FDTD method. Figure 3b–e shows the 886 nm wavelength absorption profiles of an unpassivated solar cell and solar cells with COWs of 200 nm, 400 nm, and 800 nm, respectively. The amount of absorbed power in the active layer of solar cells with the GAPL is higher than that of an unpassivated solar cell because of improved reflectivity at the rear surface of the Al_2_O_3_. Additionally, in the passivated solar cells, the active layer on the Al_2_O_3_ shows better light absorption than on the Mo surface. As the COW decreased, the absorbed power in the active layer increased because the reflectivity and coverage of Al_2_O_3_ at the rear surface increased. The average power absorbed per unit volume for the unpassivated solar cell was calculated as 1.23 × 10^12^ W/m^2^. In the solar cells with COWs of 200 nm, 400 nm, and 800 nm, the calculated average power absorption shows increments of 5.69% (1.30 × 10^12^ W/m^2^), 4.06% (1.28 × 10^12^ W/m^2^), and 1.63% (1.25 × 10^12^ W/m^2^), respectively, compared to the value of the unpassivated solar cell. The average light absorption increased nonlinearly as the area of Al_2_O_3_ at the rear surface increased. It indicates that the light absorption in the active layer increases as the nanosized COW decreases because of the improved optical path length by the diffraction grating effect of the GAPL. Therefore, as the COW decreased, the J_SC_ improved because the amount of light absorption in the thin CIGS layer increased.

**Figure 4:**
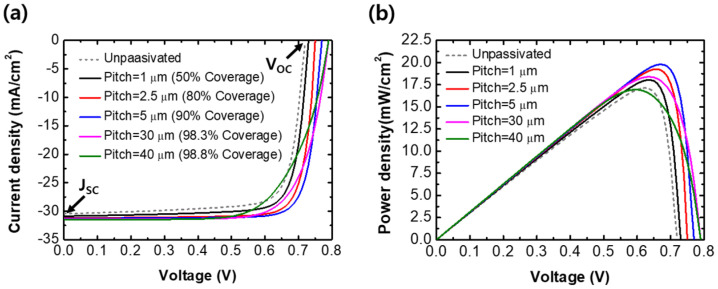
(**a**) J-V curves and (**b**) P-V curves of unpassivated and passivated thin CIGS solar cells according to the change in coverage of the GAPL with a local COW of 500 nm.

Figure 4 shows the electrical properties of the designed solar cells with the GAPL. Figure 4a shows the J-V curves calculated according to the coverage variations of the unpassivated solar cell and solar cells with a local COW of 500 nm in the GAPL. The coverage is defined as the covered ratio of the interface with Al_2_O_3_ between CIGS and Mo. The calculated J_SC_ and V_OC_ of solar cells with the GAPL were higher than those without passivation. These values increased as the GAPL coverage increased. When the pitch widened from 1 μm to 40 μm, the J_SC_ improved from 30.864 mA/cm^2^ to 31.471 mA/cm^2^. The V_OC_ increased from 0.73 V to 0.79 V. However, the maximum power of the solar cells improved as the coverage increased but decreased when the coverage was 90% or more. Figure 4b shows this tendency, which was calculated using the same structures as in Figure 4a. When the GAPL pitch was 5 μm, the maximum power density showed the highest value of 19.8 mW/cm^2^. The maximum power density decreased from 19.8 mW/cm^2^ to 16.94 mW/cm^2^ as the pitch increased from 5 μm to 40 μm. Even though the J_SC_ and V_OC_ characteristics continuously improved with increasing coverage, the power density showed the maximum value at a certain point, 90% of coverage in the suggested structure design. Thus, the FF and power conversion efficiency of solar cells with the GAPL decrease as the coverage increases.

**Figure 5:**
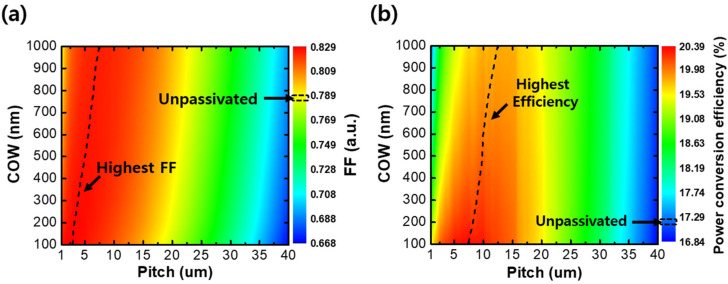
Calculated color-scaled graphs of (**a**) the fill factor (FF) and (**b**) power-conversion efficiency according to the variation in local COW and pitch of the GAPL. The dashed lines in (**a**,**b**) represent the highest FF and power-conversion efficiency, respectively.

To evaluate the FF and power-conversion efficiency distribution of the designed solar cells, we expressed these as color-scaled charts in Figure 5. Figure 5a shows the color-scaled FF distribution as a function of the local COW and pitch of the GAPL. The FF of the thin CIGS solar cell without a passivation layer was calculated as 0.78, and the highest FF was 0.83 when the COW increased in the pitch range of 3 μm to 7.5 μm (dashed line). Furthermore, the FF decreased with the pitch extension after reaching the peak value and was worse than that of the unpassivated solar cell at the pitch wider than 17.5 μm to 22.5 μm (yellow area). Figure 5b shows the distribution of the efficiency as a function of the local COW and pitch. The efficiency of the thin CIGS solar cell without the passivation layer was calculated as 17.13%. Over a wide-range pitch (≤35 μm) in the entire range of COWs, the calculated efficiency of passivated thin CIGS solar cells was higher than this value. The highest efficiency was distributed in the pitch of 7.5 μm to 12.5 μm (dashed line). The smaller the COW was, the larger the maximum efficiency was, showing a 20.38% efficiency at the 100 nm COW. However, as the pitch increased, the efficiency declined after increments to the peak value.

**Figure 6:**
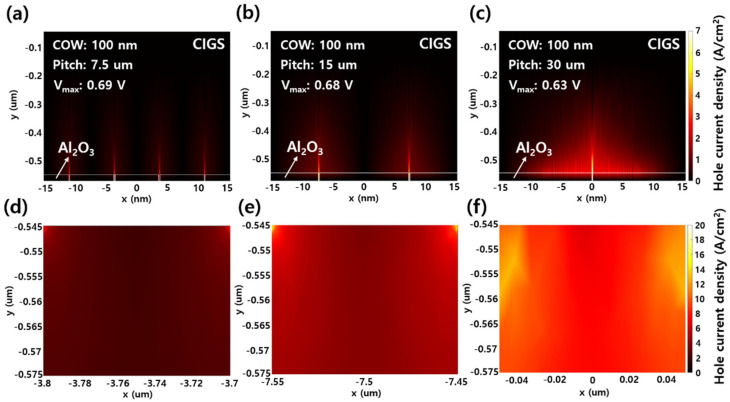
Distributions of hole current density at the maximum voltages (V_max_) for the CIGS active layer with a 100 nm COW and pitches of (**a**) 7.5 μm, (**b**) 15 μm, and (**c**) 30 μm. The magnification of a single hole in (**a**–**c**) corresponds with (**d**–**f**), respectively.

Figure 6 shows the hole current density in the active layer of thin CIGS solar cells with a 100 nm COW and pitches of (a) 7.5 μm, (b) 15 μm, and (c) 30 μm. Figure 6a–c indicates the distributions of the hole current density, and Figure 6d–f is the hole current densities in the single contact area of each structure. When the GAPL pitch was 7.5 μm, the average hole current flowing to Mo through a single contact area was calculated as 2.34 A/cm^2^ at a maximum voltage (V_max_) of 0.69 V. As the pitch increased to 15 μm and 30 μm, the average hole current density increased by 4.63 A/cm^2^ and 8.98 A/cm^2^ at the V_max_ values of 0.68 V and 0.63 V, respectively. However, the average hole current density calculated by considering the number of contact areas in each structure showed the highest value of 9.36 A/cm^2^ when the GAPL pitch was 7.5 μm. The hole current density in the GAPL pitches of 15 μm and 30 μm decreased by 1.07% and 4.6% compared to that of a pitch of 7 μm, respectively, because the long distance between the contacts increased the contact resistance [28]. The decreased hole current density lowered the J_SC_, FF, and efficiency at the pitch wider than the peak values and offset the performance advantages taken from the GAPL. In summary, extending the pitch over 12.5 μm with the sub-micron range of COWs can degrade the performance of solar cells. The simulation results were similar to practical solar cells, but showed values lower than the Shockley and Queisser limit for solar cells [41]. We will further study with experiments to reach this theoretical limit in the future. Therefore, the pitch and COW of the GAPL must be optimized to improve the performance of thin CIGS solar cells.

## 4. Conclusions

In the simulations, we investigated thin CIGS solar cells’ performance tendency by changing the COW and pitch of the GAPL. The GAPL was designed to suppress the carrier recombination rate that leads to current loss and increased light reflections at the Mo–CIGS interface. Through the GAPL structure variation, clearer increments in VOC and JSC than in the unpassivated thin CIGS solar cells were shown in the entire range of COWs and pitches. The FF and efficiency also improved in wider COW ranges, with half and three-quarters of pitch variations, respectively. When the COW and pitch were 100 nm and 3 μm, the FF showed a maximum value of 0.83. In addition, the maximum value of the efficiency was calculated to be 20.38% at the COW of 100 nm and pitch of 7.5 μm. This indicates that it is possible to design high-efficiency thin CIGS solar cells with an optimized passivation structure that minimizes current loss because of carrier recombination and poor reflectivity of Mo. Considering these results, the PV designers can realize the higher efficiency of thin CIGS solar cells for PV generators and photosensitive sensors.

## Data Availability

The data presented in this study are available on request from the corresponding author.
